# Antiseptic Transfusion of Human Blood

**Published:** 1880-01-20

**Authors:** William Macewen


### *ANTISEPTIC TRANSFUSION OF HUMAN BLOOD.*

BY WILLIAM MACEWEN, M.D.

The following case of transfusion of human blood is worthy of record, on account of the antiseptic precautions which were adopted, and the complete success of the operation. The patient was a man, twenty-three years of age, on whom lithotomy was performed for the removal of a large spiked oxalate-of-lime calculus. There was little bleeding as an immediate sequent of the operation, and it was completely arrested before he left the table. Half an hour after having been put to bed, the house-surgeon found him to be very comfortable and in a good general state ; there was then only slight staining on the sheet under the pelvis. Two hours and a half after he was found to be in a state of complete depletion, from a profuse secondary hemorrhage which had ensued. The wound was at once plugged. Notwithstanding every attempt to resuscitate him by stimulating drinks, enemata, etc., it was evident, at the end of three-quarters of an hour, that ground was being fast lost, and it was clear to all present that, if his life was to be saved, something more radical was necessary. Transfusion of blood was proposed. A patient who suffered from injury to the right great toe, and who otherwise was strong and healthy, after being apprised of the slight risk he ran in giving a portion of his blood, freely offered it to his fellow.

The lithotomy patient was in the following state. He was semi-insensible, could not speak, pulse at the wrist imperceptible, surface of the body blanched and bedewed with a cold perspiration, the lips cream-colored, and the conjunctival vessels no longer visible. Occasionally he gave a restless feeble toss, accompanied by a deep inspiration.

An attempt to find one of the large veins on the right arm being unsuccessful, the median cephalic of the left was chosen, and half an inch of its length exposed. An assistant was desired to place the finger of one hand to the distal side of the exposed vein, and to maintain pressure on that part thoroughout the operation, so as to prevent loss of blood ; and with a finger of the other hand, placed on the proximal side, about half an inch above the part selected for opening the vessel, he was to occlude the vein when required. The arm was held well up, so as to empty it of any blood which it might contain. It was also maintained considerably above the level of the patient's body, for three reasons: First, to facilitate the flow of the transfused blood into the trunk; secondly, to prevent the entrance of air into the body, as the syringe would, with the arm in this position, be necessarily held perpendicularly, with the nozzle downwards, and all contained air would remain at the top of the instrument; and thirdly, to enable any air to escape which might be in the space intervening between the opening in the vein and the occluding finger of the assistant on the proximal side. The vein was then opened. Then phlebotomy was performed on the healthy man, the blood being received into a small warm carbolised vessel, from which it was at once drawn into a warm carbolised three-ounce syringe, having a narrow nozzle. When full it was inverted and the piston pressed, so as to expel any air, and the nozzle was then introduced into the vein. A quantity of blood was first injected into the space in the vein, between the occluding finger on the proximal side and the opening in the vein itself. When this was done the pressure on the proximal side was removed, and the contents of the syringe were slowly injected, until only a couple of drachms remained. The pressure of the assistant's finger was again applied, and the syringe removed. It was then washed in 1 to 80 carbolised watery solution, recharged, and the blood introduced as before. The tin into which the blood flowed was kept free from clot, and several times a fresh cup was substituted. The arm from which the blood was drawn. as well as that into which it was injected, were kept constantly under the spray, and the blood itself, from the time it left the one arm until it was injected into the other, was either exposed to the carbolised spray or in contact with carbolised instruments; so that the whole transfusion was thoroughly antiseptic.

With the exception of the transfusion being performed antiseptically, the other details of the operation were nearly the same as those adopted by Mr. Lister in a case in which he performed transfusion, while I acted as one of the house-surgeons in the Royal Infirmary. That case was reported by me in the Glasgow Medical Journal for November, 1869.

Just before the transfusion was begun several of the house-surgeons hinted that the patient had "slipped away." His heart, however, was heard to respond, and the blood was injected. Shortly after the transfusion the gentleman who had his finger over the radial said that, from being imperceptible, it had returned gradually, and had increased until it was distinctly felt. Half an hour after, the face had assumed a slight redness, and heat began to be restored to the surface of the body. There were no rigors after the transfusion. Without entering into further detail. it may be said that he slowly but perfectly recovered, and is now a strong, healthy man. He was shown at the Pathological and Clinical Society nine months after the operation, and is still quite well and at work.—*London Lancet.*

---

THE "aleximorhygiastikon" is a patent pocket inhaler advertised in the London medical journals; but what people who want it are expected to say when they go to the apothecary's shop is not stated.

